# Supplementary material for: Medical slice transformer for improved diagnosis and explainability on 3D medical images with DINOv2
Source: Sci Rep. 2025 Jul 4;15:23979. doi: 10.1038/s41598-025-09041-8 (PMC12227771; doi:10.1038/s41598-025-09041-8)
Supplement: Supplementary file 1 — Supplementary Material 1 [file 41598_2025_9041_MOESM1_ESM.pdf]

Medical Slice Transformer for Improved Diagnosis and Explainability on 3D Medical Images with DINOv2

Gustav Müller-Franzes[1*], Firas Khader[1], Robert Siepmann[1], Tianyu Han[1], Jakob Nikolas Kather[2,3,4], Sven Nebelung[1†], Daniel Truhn[1†]

[1]Department of Diagnostic and Interventional Radiology, University Hospital Aachen, Aachen, Germany
[2]Else Kroener Fresenius Center for Digital Health, Technical University Dresden, Dresden, Germany
[3]Department of Medicine I, University Hospital Dresden, Dresden, Germany
[4]National Center for Tumor Diseases (NCT), University Hospital Heidelberg, Heidelberg, Germany
[*]Corresponding Author: gumueller@ukaachen.de
[†]Contributed equally

Supplementary Material

Saliency Maps Ablation Experiments

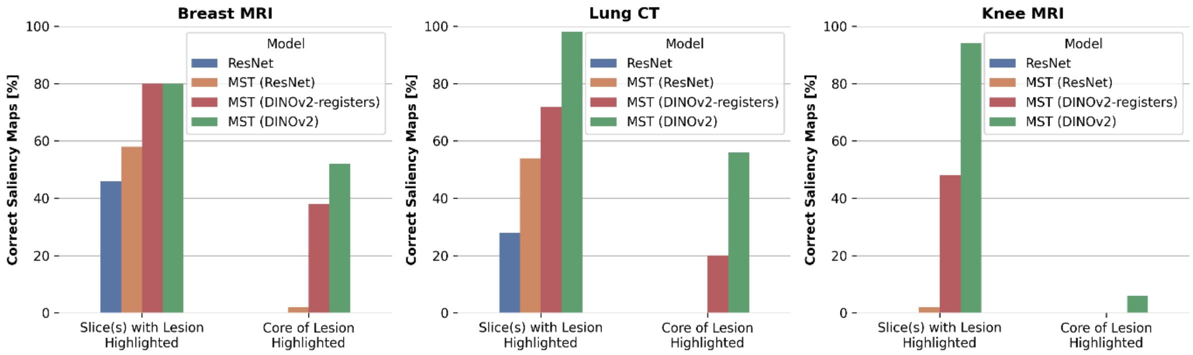

Figure S1: Quantification of Saliency Map-Lesion-Correspondence as a Function of Imaging Dataset and Model Architecture. Percentages of (blinded) radiologist evaluation in terms of slice correctness (“Does the saliency map highlight the slice(s) containing the lesion?” - yes/no) and lesion correctness (“Does the saliency map accurately point to the core of the lesion?” - yes/no).

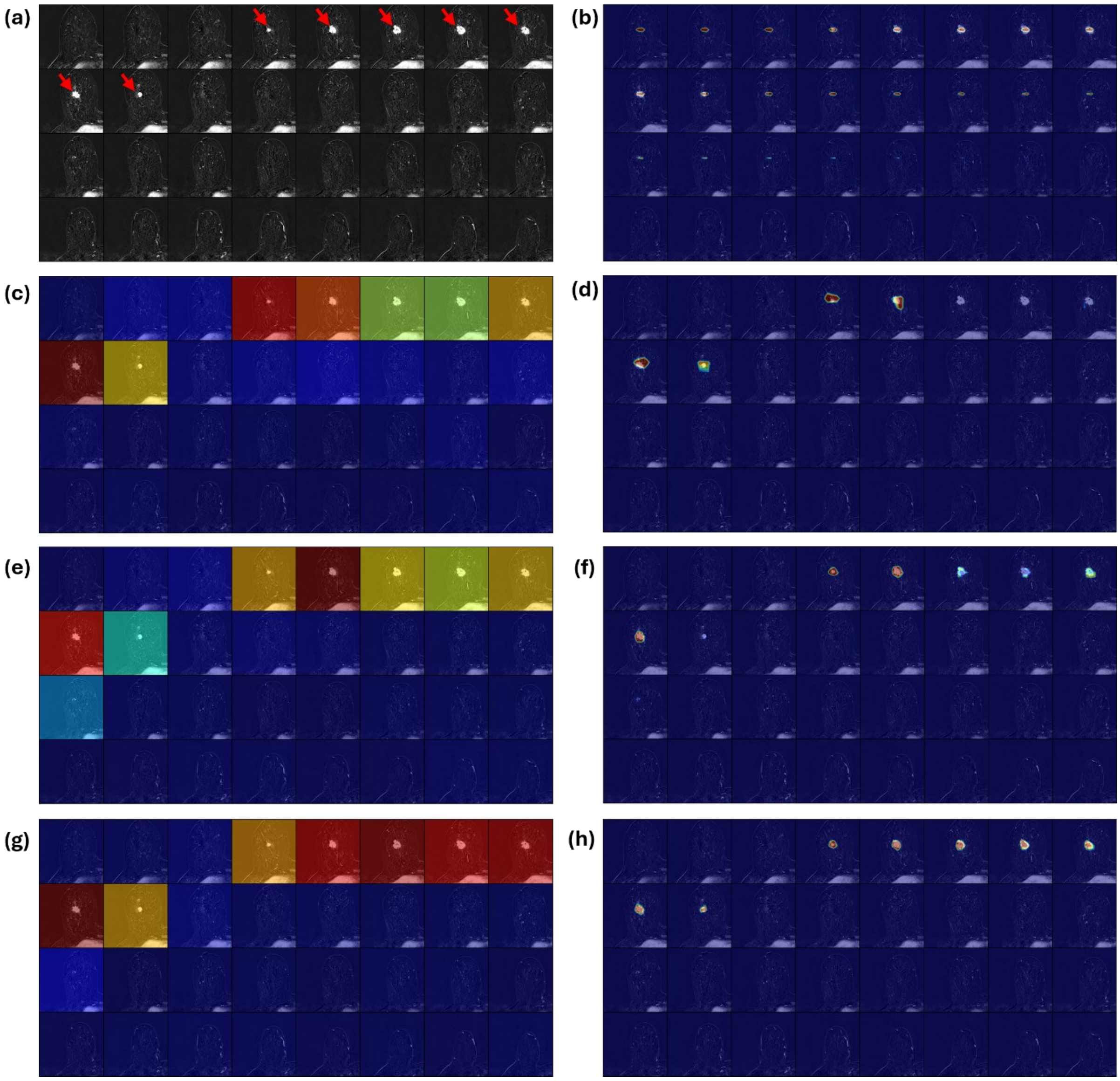

Figure S2: Saliency Maps as a Function of Model Architecture on the Breast MRI Dataset. (a) Consecutive axial slices of an MRI scan of the right side of the breast showing subtraction images with a malignant lesion highlighted by red arrows. (b) The saliency maps of the conventional 3D ResNet are spread across all provided sections of the dataset and highlight attention on slices without the lesion. The color coding toward blue indicates low attention, while the spectrum toward red indicates high attention. In contrast, the MST framework focuses on the lesion when using ResNet (c), DINOv2-registers (e), or DINOv2 (g) as an image encoder. The combined attention map results from two attention mechanisms, i.e., the Slice Transformer's attention to specific slices and the within-slice attention to specific patches when using ResNet (d), DINOv2-registers (f) or DINOv2 (h) as image encoder. The precision of lesion localization improves as a function of image encoders, with DINOv2 showing the highest precision.

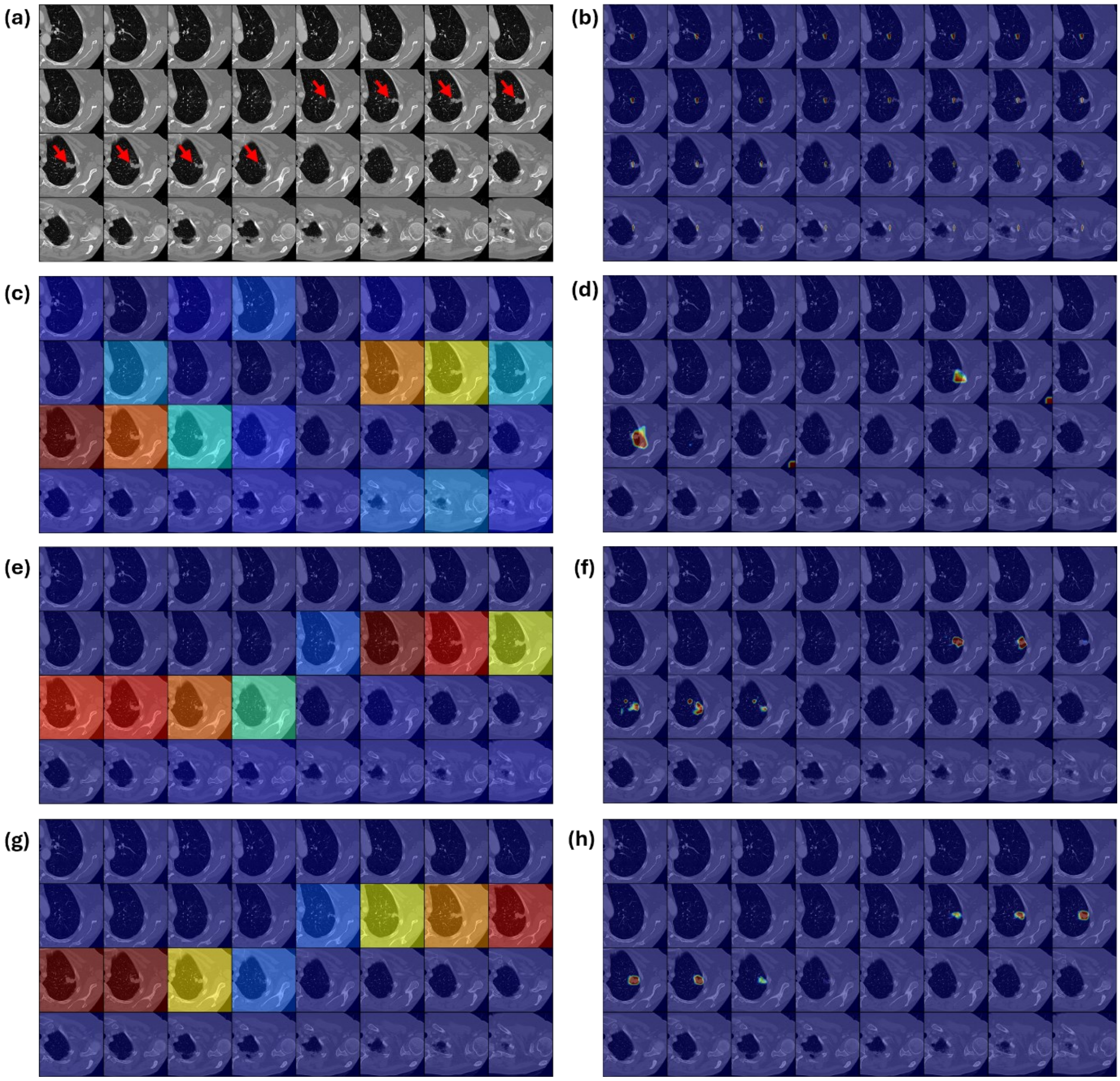

Figure S3: Saliency Maps as a Function of Model Architecture on the Lung CT Dataset. (a) Consecutive axial slices of a left sided lung CT scan centered around a large pulmonary nodule highlighted by red arrows. (b-h) Image organization as in **Figure S2**.

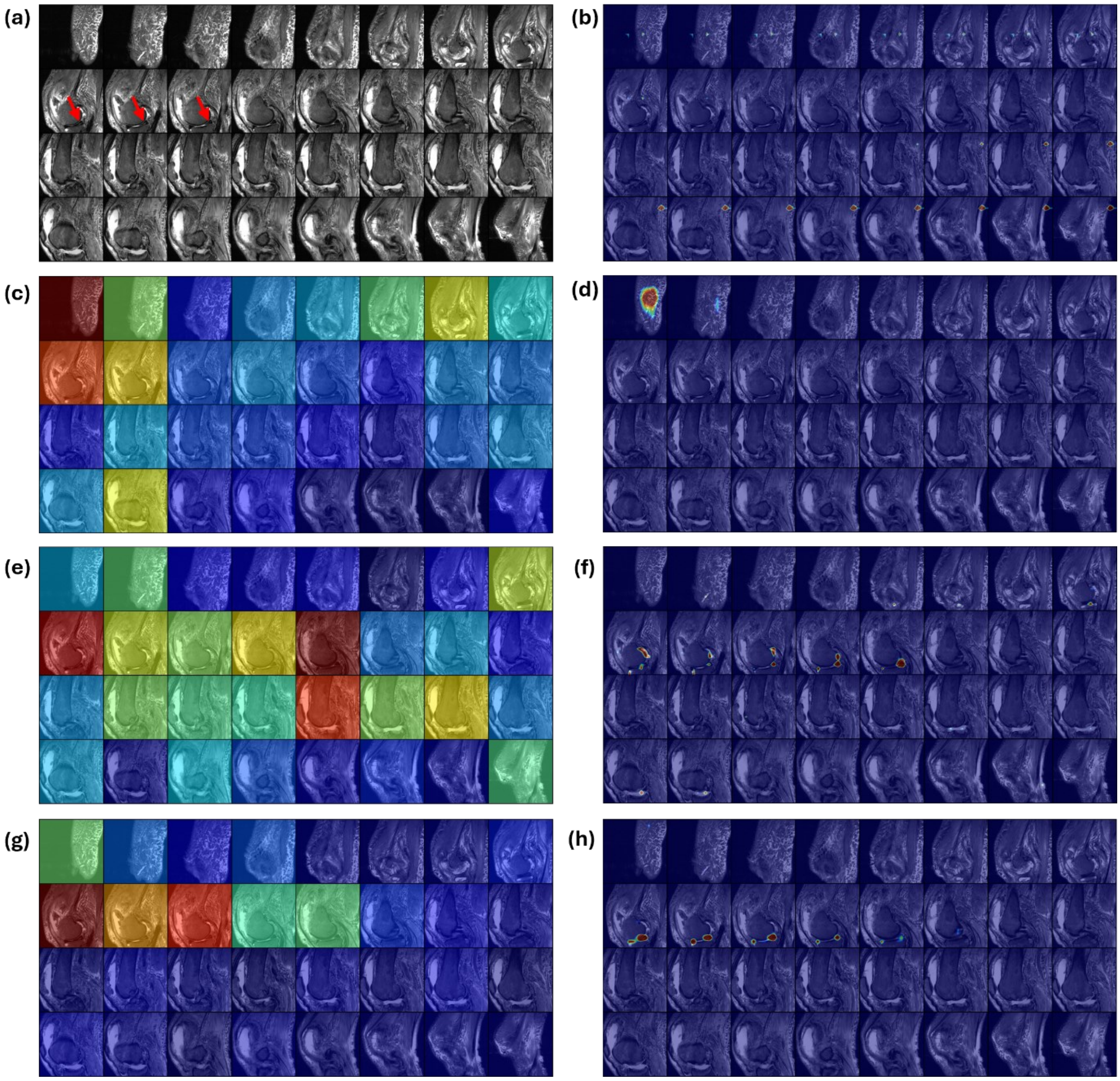

Figure S4: Saliency Maps as a Function of Model Architecture on the Knee MRI Dataset. (a) Consecutive sagittal slices of an MRI scan of a knee with meniscus tear highlighted by red arrows. (b-h) Image organization as in **Figure S2**.

Table S1: Classification Performance as a Function of the ResNet Variant and Dataset. Results are shown as mean accuracy ± standard deviation. The best-performing model for each dataset is highlighted in bold.

	Breast MRI	Chest CT	Knee MRI
**ResNet50**	**0.91±0.02**	**0.92±0.02**	0.69±0.05
**ResNet18**	0.91±0.02 (P=0.63)	0.91±0.02 (P=0.42)	0.63±0.05 (P=0.18)
**ResNet101**	0.90±0.02 (P=0.40)	0.92±0.02 (P=0.97)	**0.70±0.05** (P=0.72)
